# Targeting the prohibitin scaffold-CRAF kinase interaction in RAS-ERK-driven pancreatic ductal adenocarcinoma

**DOI:** 10.1186/1476-4598-13-38

**Published:** 2014-02-25

**Authors:** Zhou Luan, Ying He, Mohamed Alattar, Zhishui Chen, Fan He

**Affiliations:** 1 Institute of Organ Transplantation, Tongji Hospital, Tongji Medical College, Huazhong University of Science and Technology, Wuhan, China; 2 Key Laboratory of Ministry of Health and Key Laboratory of Ministry of Education, Wuhan, China; 3 Department of Ophthalmology, Shanghai First Hospital, Shanghai Jiao Tong University School of Medicine, Shanghai, China; 4 Department of Cardiothoracic Surgery, Zagazig University Hospital, Alsharkia, Egypt

**Keywords:** Prohibitin, Rocaglamide, Pancreatic ductal adenocarcinoma, Targeted therapy

## Abstract

**Background:**

Robust ERK1/2 activity, which frequently results from KRAS mutation, invariably occurs in pancreatic ductal adenocarcinoma (PDAC). However, direct interference of KRAS signaling has not led to clinically successful drugs. Correct localization of RAF is regulated by the scaffold protein prohibitin (PHB) that ensures the spatial organization between RAS and RAF in plasma membranes, thus leading to activation of downstream effectors.

**Methods:**

PHB expression was analyzed in human pancreatic cancer cell lines, normal pancreas, and PDAC tissue. Furthermore, genetic ablation or pharmacological inhibition of PHB was performed to determine its role in growth, migration, and signaling of pancreatic cancer cells *in vitro* and *in vivo*.

**Results:**

The level of PHB expression was crucial for maintenance of oncogenic ERK-driven pancreatic tumorigenesis. Additionally, rocaglamide (RocA), a small molecular inhibitor, selectively bound to PHB with nanomolar affinity to disrupt the PHB-CRAF interaction by altering its localization to the plasma membrane. Consequently, there was an impairment of oncogenic RAS-ERK signaling, thereby blocking *in vitro* and *in vivo* growth and metastasis of pancreatic cancer cells that were addicted to RAS-ERK signaling. More importantly, RocA treatment resulted in a significant increase of the lifespan of tumor-bearing mice without any detectable toxicity.

**Conclusions:**

Blockade of the PHB scaffold-CRAF kinase interaction, which is distinct from direct kinase inhibition, may be a new therapeutic strategy to target oncogenic ERK-driven pancreatic cancer.

## Background

The almost universal lethality of pancreatic ductal adenocarcinoma (PDAC) has led to intensive study of the genetic mutations responsible for its initiation and progression [1-3]. The most common oncogenic mutations associated with all PDAC stages occur in the KRAS gene, indicating that this gene is the primary initiator of PDAC [4]. However, RAS is an intractable therapeutic target and RAS inhibitors have not been successful in clinical trials. Therefore, targeting downstream kinases in the pathway such as RAF and MEK may be a new approach [5,6]. Unfortunately, the structures of the catalytic domains of various kinases are highly similar and many “specific” inhibitors target multiple kinases rather than their intended target [7]. Additionally, cancer cells rapidly acquire resistances against kinase inhibitors. Thus, novel therapeutics targeting regions outside the kinase domain have become much more necessary for components of the RAS-RAF-ERK pathway.

Intracellular scaffold proteins mediate protein-protein interactions as well as spatial and temporal regulation to generate signal specificity, which ultimately controls cellular behavior [8,9]. Prohibitin (PHB), a flagship member of the Band-7 family of proteins, is highly conserved, ubiquitously expressed, and localizes to the mitochondria, cytosol, nucleus, and plasma membrane [10-13]. Notably, PHB is a scaffold protein required for the interaction between RAS and RAF at the plasma membrane, thus leading to RAS-mediated activation of RAF and downstream activation of the ERK pathway [14]. Intriguingly, PHB-silenced HeLa cells exhibit reduced spreading and increased intercellular adhesion, forming tiny islands of densely packed cells [15]. We observed that the pancreatic cancer cell line Capan-2 exhibits similar tiny islands of densely packed cells. Therefore, we hypothesized that deficient PHB expression may exist in Capan-2 cells. In addition, whether PHB plays any role in RAS-ERK-driven pancreatic cancer remains undetermined.

Rocaglamide (RocA), a naturally occurring compound, has a unique cyclopenta [b] benzofuran skeleton and is isolated from the medicinal plants belonging to genus *Aglaia* (family *Meliaceae*) [16], which are traditionally used in folk medicine for the treatment of coughs, injuries, asthma, and inflammatory skin diseases. More recently,

Polier *et al.*[17] carried out affinity chromatography-coupled mass spectrometry to identity PHB as the direct target of RocA in leukemic cells. Importantly, they also revealed the mechanism in which binding of RocA to PHB prevents CRAF-PHB interactions, thus leading to impaired ERK1/2 activation in leukemic cells. Therefore, RocA may be used to target protein-protein interactions rather than the catalytic kinase domain.

In the present study, we unravel a new therapeutic paradigm to inhibit RAS-driven pancreatic tumors by blocking the interactions of PHB scaffold-CRAF kinase. Furthermore, RocA suppresses ERK activity and blocks *in vitro* and *in vivo* growth and metastasis of pancreatic cancer cells that are addicted to the ERK pathway.

Thus, the regulation of RAS-RAF-ERK pathway by targeting the PHB-CRAF interaction introduces a novel potential therapeutic approach for ERK-driven pancreatic cancer.

## Results

### Expression and localization of PHB in pancreatic cancer cells and tissue

**Figure 1:**
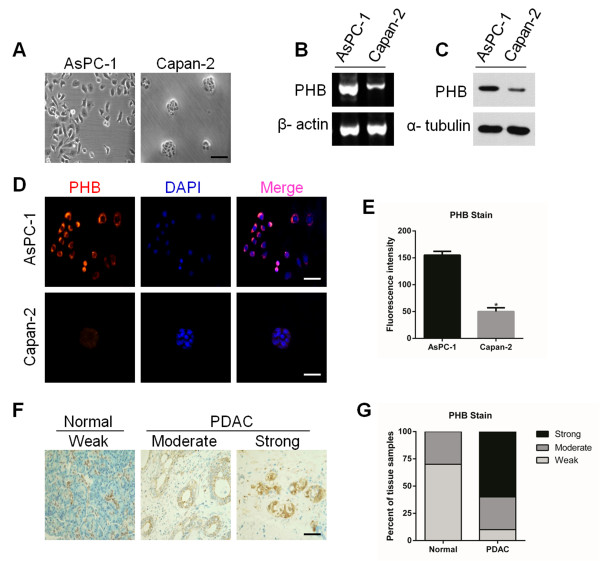
**Expression of PHB in pancreatic cancer cells and tissue. A**. Morphology of AsPC-1 and Capan-2 cells. Scale bar, 25 μm. **B**. RT-PCR analysis of PHB mRNA expression in AsPC-1 and Capan-2 cells. β-actin was used as a control. **C**. Immunoblot analysis of PHB protein in AsPC-1 and Capan-2 cells. α-tubulin was used as a control. **D**. Confocal microscopic images of the localization of PHB (red) in AsPC-1 and Capan-2 Cells. Nuclei were counterstained with DAPI. Scale bars, 25 μm. **E**. Quantification of the fluorescence intensity in **(D)**. Values are the means ± SD of triplicate samples, *P < 0.01. **F**. Representative images of PHB expression in PDAC and normal epithelial tissues. Scale bar, 50 μm. **G**. Quantification of PHB expression in PDAC and normal pancreas tissues. Tumors were blindly scored based on the strength of PHB staining (n = 11 normal tissue samples and n = 46 PDAC tissue samples). Results are representative of three independent experiments.

To investigate the role of PHB in pancreatic cancer cells, we first chose two human pancreatic cancer cell lines, AsPC-1 (high malignancy) and Capan-2 (low malignancy). Interestingly, AsPC-1 cells grew as single cells (Figure 1A, left), whereas Capan-2 cells exhibited tiny islands of densely packed cells (Figure 1A, right). Additionally, AsPC-1 cells exhibited much higher growth and migration capacities than those of Capan-2 cells (Additional file 1: Figure S1A,B). RT-PCR showed a difference in PHB mRNA expression levels, revealing higher expression in AsPC-1 cells than that in Capan-2 cells (Figure 1B and Additional file 1: Figure S2A). In agreement with RT-PCR data, immunoblot analysis also demonstrated high expression of PHB protein in AsPC-1 cells, but little expression in Capan-2 cells (Figure 1C and Additional file 1: Figure S2B). Intriguingly, localization of PHB in AsPC-1 cells was mainly in the plasma membrane and cytosol, whereas its localization was in Capan-2 cells (Figure 1D,E). This result indicated that the observed phenotypes may correlate with the expression and localization of PHB protein. Therefore, AsPC-1 cells were chosen to investigate the biological properties of PHB in pancreatic cancer both *in vitro* and *in vivo*.

We next assessed PHB expression in pancreatic tissue. PHB protein was weakly expressed in 63.6% of normal pancreas samples (n = 11) (Figure 1F,G). However, PHB protein was strongly expressed in 58.7% of PDAC samples (n = 46) (Figure 1F,G and Additional file 1: Figure S3). Taken together, these results show that PHB, which becomes more pronounced with pancreatic cancer malignancy, may serve as a therapeutic target in pancreatic cancer.

### PHB is indispensable for EGF-induced ERK activation in pancreatic cancer cells

**Figure 2:**
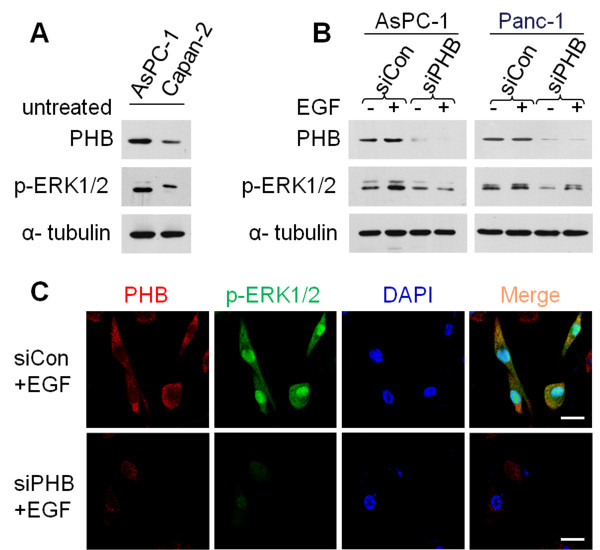
**PHB is indispensable for EGF-induced ERK activation in pancreatic cancer cells. A**. Immunoblot analysis of PHB and p-ERK1/2 levels in AsPC-1 and Capan-2 cells. α-tubulin was used as a loading control. **B**. AsPC-1 and Panc-1 cells were transfected with PHB-specific (siPHB) or control (siCon) siRNAs. At 48 h post-transfection, cells were treated with EGF (50 ng/ml) for 15 min and then subjected to immunoblot analysis using the indicated antibodies. p-ERK1/2 was detected as an activation marker and α-tubulin as the loading control. **C**. Confocal microscopic images of the localization of PHB (red) and p-ERK1/2 (green). AsPC-1 cells were transfected with PHB-specific (siPHB) or control (siCon) siRNAs. After 48 h of transfection, cells were stimulated with EGF (50 ng/ml) for 15 min. AsPC-1 cells were incubated with antibodies specific for PHB and p-ERK1/2. Nuclei were counterstained with DAPI. Scale bars, 10 μm. Results are representative of three independent experiments.

The duration of ERK activity is a crucial factor in diverse biological processes that determine cell fate decisions [18]. ERK is phosphorylated and activated by MEK in response to growth factor stimulation, and then activated ERK phosphorylates and activates nuclear targets to up-regulate immediate-early genes [19]. Therefore, we determined the expression levels of p-ERK1/2 in AsPC-1 and Capan-2 cells. Intriguingly, the phosphorylation status of ERK2 was much higher than that of ERK1 in AsPC-1 cells, and this phenomenon was completely converse in Capan-2 cells (Figure 2A). This observation suggests distinct roles of ERK1 and ERK2 in the regulation of cell behavior in AsPC-1 and Capan-2 cells.

To test whether PHB is required for the ERK pathway, we validated a siRNA against PHB (siPHB) in AsPC-1 and Panc-1 cells by quantitative real-time PCR. The results showed that siPHB reduced the PHB mRNA level by about 80% compared with that using control siRNA (siCon) (Additional file 1: Figure S4A,B). Furthermore, we checked the phosphorylation status of ERK1/2 in siPHB-transfected AsPC-1 and Panc-1 cells. As expected, stimulation of AsPC-1 cells with epidermal growth factor (EGF) caused an increase of ERK1/2 phosphorylation (Figure 2B,C), whereas silencing of PHB expression strongly suppressed the EGF-induced phosphorylation of ERK (Figure 2B,C). This finding suggested specific involvement of PHB in the RAS-RAF-ERK pathway. Moreover, a similar result was obtained in Panc-1 cells (Figure 2B), indicating general inhibition of ERK activation by PHB depletion. Thus, these results clearly indicate that PHB is required for EGF-induced ERK1/2 activation in pancreatic cancer cells.

### RocA disrupts the ERK pathway by targeting the CRAF-PHB interaction in AsPC-1 cells

**Figure 3:**
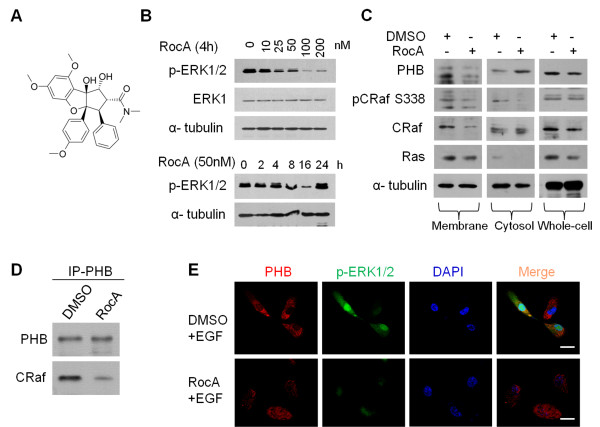
**RocA disrupts the RAS-ERK pathway by targeting the CRaf-PHB interaction in AsPC-1 cells. A**. Chemical structure of RocA. **B**. AsPC-1 cells were treated with RocA at various concentrations or time periods as indicated and then the activation status of ERK was examined by immunoblot analysis. ERK1 and α-tubulin were used as controls. **C**. Analysis of PHB-CRAF membrane localization in AsPC-1 cells in the presence of RocA or DMSO. AsPC-1 cells were treated with RocA (100 nM) or DMSO for 16 h. Whole cell lysates as well as cell membrane and cytosol fractions were prepared and then subjected to immunoblot analysis with the indicated antibodies. Phospho-CRAF (pSer338) was used as marker of activity and α-tubulin as the loading control. **D**. Immunoprecipitation of PHB protein and associated CRAF from AsPC-1 cells following 4 h of treatment with RocA (100 nM) or DMSO. **E**. Confocal microscopic images of the localization of PHB (red) and p-ERK1/2 (green). AsPC-1 cells were treated with RocA (100 nM) or DMSO for 4 h and then stimulated with EGF (50 ng/ml) for 15 min. AsPC-1 cells were incubated with antibodies specific for PHB or p-ERK1/2. Nuclei were counterstained with DAPI. Scale bars, 10 μm. Results are representative of three independent experiments.

The oncogenic RAS-ERK pathway is a key node for cellular proliferation signals and has been the focus of substantial drug discovery efforts in many cancers [20-23]. A previous study has indicated that RocA suppresses the ERK pathway in leukemic cells [24]. To confirm that the anti-tumor effect of RocA is indeed caused by suppression of the ERK pathway, we examined the effect of RocA on ERK activity in AsPC-1 cells (Figure 3E). The results showed significant dose-dependent inhibition of the phosphorylation status of ERK1/2 (Figure 3B). Importantly, RocA showed very strong time-dependent suppression of ERK1/2 activities (Figure 3B).

PHB was previously shown to be required for membrane association and activation of CRAF [15]. Therefore, we examined whether RocA affects PHB-CRAF membrane association in AsPC-1 cells. To this end, cell membrane and cytosol fractions were prepared from AsPC-1 cells treated with Roc-A or DMSO to analyze the localization of PHB and CRAF. Immunoblot analysis showed significant reduction of CRAF, particularly phosphorylated CRAF (pSer338), in the membrane fraction after RocA treatment (Figure 3C). Notably, RocA also significantly reduced the levels of PHB in the membrane fraction, indicating that binding of RocA to PHB may also interfere with PHB membrane association. However, RocA did not influence membrane localization of RAS (Figure 3C). Indeed, immunoprecipitation analysis suggested that RocA substantially decreased the amounts of total CRAF bound to PHB in AsPC-1 cells (Figure 3D). Notably, confocal microscopic analysis showed that treatment of AsPC-1 cells with 100 nM RocA for 4 h led to a loss of plasma membrane localization and random redistribution of PHB (Figure 3E). This observation indicates that inhibition of the PHB-CRAF interaction by RocA leads to the loss of spatial organization of PHB in AsPC-1 cells. Collectively, these results further demonstrate that RocA blocks the RAS-CRAF-ERK signaling pathway by disruption of the PHB-CRAF interaction in pancreatic cancer.

### RocA mimics the effect of PHB knockdown on epithelial-mesenchymal transition (EMT) markers and reverses the EMT phenotype in AsPC-1 cells

**Figure 4:**
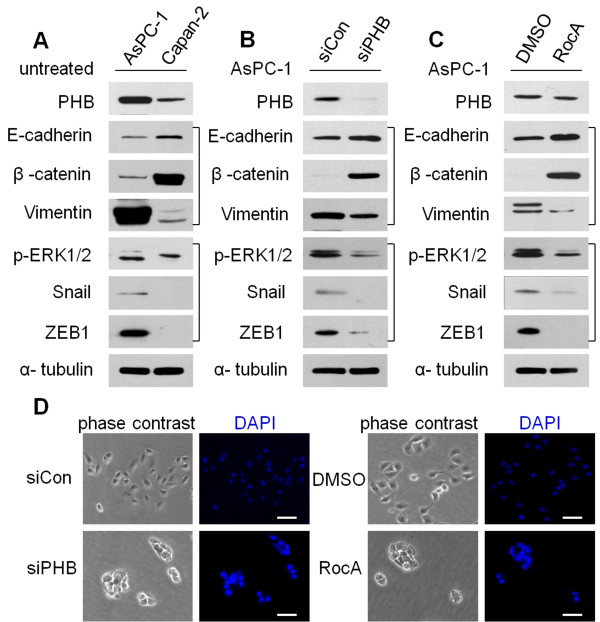
**RocA mimics PHB-knockdown effects on EMT markers and reverses EMT in AsPC-1 cells. A**. AsPC-1 and Capan-2 cells were lysed and then subjected to immunoblot analysis with the indicated antibodies. **B**. AsPC-1 cells were transfected with PHB-specific (siPHB) or control (siCon) siRNAs. After 48 h of transfection, the cells were lysed and subjected to immunoblot analysis with the indicated antibodies. **C**. AsPC-1 cells were treated with RocA (100 nM) or DMSO for 16 h, and then subjected to western blot analysis with the indicated antibodies. **D**. Morphological changes of PHB-knockdown AsPC-1 cells **(B)** and RocA-treated AsPC-1 cells **(C)** treated with control siRNAs (siCon) or DMSO. Scale bars, 25 μm. Results are representative of three independent experiments.

The oncogenic RAS-RAF-ERK pathway confers epithelial cells with critical motile and invasive capacities during carcinoma progression, often by promotion of EMT [25,26]. To further investigate the role of PHB in EMT, the effects of PHB siRNA and RocA on EMT markers were assayed in AsPC-1 cells. First, we detected EMT markers in AsPC-1 and Capan-2 cells (Figure 4A). Knockdown of PHB in AsPC-1 cells by siRNA resulted in upregulation of E-cadherin and β-catenin and downregulation of vimentin (Figure 4B). Similar to the effect of PHB knockdown, treatment of AsPC-1 cells with RocA showed the same results (Figure 4C).

Activated ERK2 directly phosphorylates Snail, leading to nuclear accumulation, reduced ubiquitylation, and an increased protein half-life of Snail, and then promotion of breast cancer cell invasion and migration *in vitro* and metastasis *in vivo*[27]. Another study has shown clear increases of ZEB1 and ZEB2 protein levels by ERK2 but not ERK1 [28]. To further investigate the molecular basis of ERK-regulated EMT, we detected the levels of Snail1, ZEB1, and transcription factors known to regulate EMT which act downstream of ERK1/2. Interestingly, we observed similar results in PHB-silenced and RocA-treated AsPC-1 cells (Figure 4B,C).

AsPC-1 cells lacking PHB expression showed defective migration (Figure 4D), indicating that the formation of clusters is the consequence of reduced motility of cells that lack high levels of PHB. Notably, AsPC-1 cells treated with RocA formed cell clusters similar to those formed by cells with reduced PHB expression (Figure 4D). Taken together, RocA mimics the effect of PHB knockdown on EMT marker expression and reverses the EMT phenotype in AsPC-1 cells.

### RocA selectively diminishes the viability of PHB-dependent pancreatic cancer cells *in vitro* and inhibits their migration *in vitro* and *in vivo*

**Figure 5:**
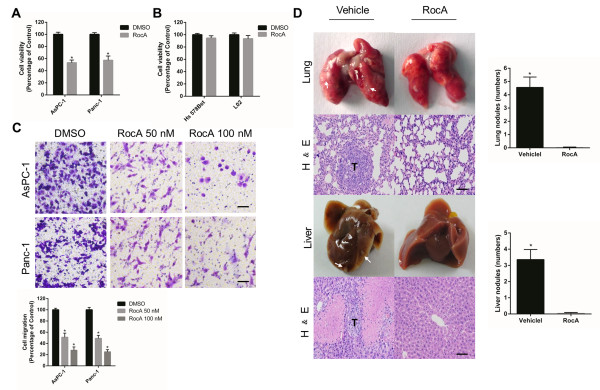
**RocA selectively diminishes the viability and migration of KRAS-mutated pancreatic cancer cells. A**. Viability of DMSO- and RocA-treated AsPC-1 and Panc-1 cells. Values are the means ± SD of triplicate samples, *P < 0.01. **B**. Viability of DMSO- and RocA-treated Hs 578Bst and L02 cells. Values are the means ± SD of triplicate samples, *P < 0.01. **C**. Transwell migration assays of AsPC-1 and Panc-1 cells treated with RocA (50 or 100 nM). The plot shows the quantification of cells that passed through the filters relative to DMSO that was set to 100%. Values are the means ± SD of triplicate samples, *P < 0.01. Scale bars, 25 μm. **D**. Effect of RocA on pancreatic cancer metastasis in an orthotopic xenograft model. AsPC-1 cells were orthotopically injected into the pancreas of mice (n = 6) as described in the Methods. At 1 week post-implantation, RocA (5.0 mg/kg, n = 3) or the vehicle (1% DMSO in olive oil, n = 3) was administrated via daily intraperitoneal injection for 4 weeks. Then, the lungs and livers of mice were collected and processed for H&E staining. The number of tumor foci was counted in the lungs and livers of sacrificed mice. Arrows indicate tumor foci. T, tumor. Values are the means ± SD of triplicate samples, *P < 0.01. Scale bars, 50 μm.

To characterize the action of RocA on pancreatic cancer cell growth, AsPC-1 and Panc-1 cells were treated with RocA (100 nM) or DMSO for 16 h and then applied to CCK-8 assays. RocA markedly impaired the growth of AsPC-1 and Panc-1 cells without affecting Hs 578Bst or L02 cells as controls (Figure 5A,B). Interestingly, Capan-2 cells did not show any detectable toxicity in the presence of RocA (Additional file 1: Figure S5), suggesting deficient expression of PHB in Capan-2 cells may rescue the effects of RocA. Additionally, RocA impaired the migration of AsPC-1 and Panc-1 cells (Figure 5C).

To investigate the effect of RocA on metastasis, we established an orthotopic xenograft model in mice using AsPC-1 cells. At 1 week after orthotopic implantation of AsPC-1 cells into severe combined immunodeficient (SCID) mice, RocA (5 mg/kg body weight) was administrated via intraperitoneal injection daily for 3 weeks. As a result, treatment with RocA significantly suppressed cancer metastasis to the lung and liver in mice (Figure 5D). Histological analysis of the lung and liver revealed that dissemination of cancer cells was absent in tissue sections from RocA-treated mice, but an abundance of cancer cells were observed in vehicle-treated mice (Figure 5D). Comparison of the survival curve of RocA-treated mice with that of vehicle-treated mice showed that RocA treatment significantly prolonged the survival of tumor-bearing mice (Additional file 1: Figure S6A,B). Taken together, RocA impairs the migration of pancreatic cancer cells *in vitro* and *in vivo*.

### RocA suppresses *in vivo* growth of tumor xenografts

**Figure 6:**
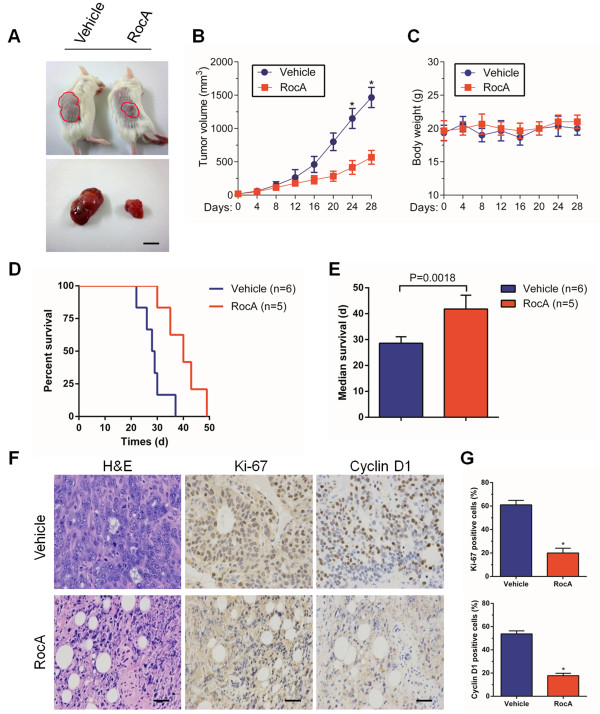
**RocA suppresses *in vivo *tumor growth in a xenograft model.** Subcutaneously established AsPC-1 cell-derived tumors in SCID mice were treated with the vehicle (1% DMSO in olive oil, n = 6) or RocA (5.0 mg/kg, n = 5) via daily intraperitoneal injection for 48 days. The tumor volume **(A, B)** and body weight **(C)** were monitored twice per week for 28 days. Values are the means ± SD of triplicate samples, *P < 0.01. Scale bar, 1 cm. **D**. Survival of mice with established tumor burden randomized to receive RocA or vehicle by intraperitoneal injection. **E**. Median survival of the mice in **(D)**. Statistical significance was calculated by the log-rank test. Data are shown as the means ± SD. **F**. Histological analysis of tumors from **(A, B)** was performed by H&E staining and examining Ki-67 and cyclin D1 expression to compare cell proliferation in RocA- and vehicle-treated tumors. Scale bars, 50 μm. **G**. Quantification of Ki-67- and cyclin D1-positive cells in **(F)**. Values are the means ± SD of triplicate samples, *P < 0.01.

To further evaluate the anti-tumor activity of RocA, we administered RocA to SCID mice bearing subcutaneous AsPC-1 tumor cell xenografts and monitored the tumor growth rate. RocA was administrated by intraperitoneal injection once per day. As a result, RocA significantly suppressed tumor growth compared with that in the control group. Tumor volumes in the RocA-treated group were 37 ± 8% of those in the control group (Figure 6A,B). Intriguingly, RocA treatment neither caused any loss of body weight nor exhibited apparent signs of toxicity in mice during the treatments (Figure 6C), suggesting that RocA is generally well tolerated *in vivo*. Moreover, although RocA-treated mice eventually died from the pancreatic tumors, treatment with RocA significantly extended their lifespan compared with that of vehicle treatment (Figure 6D,E).

Next, we investigated the effect of RocA on cell proliferation *in vivo* by hematoxylin and eosin (H&E) staining and examining Ki-67 and cyclin D1 expression in tumor tissues harvested from vehicle- and RocA-treated mice. H&E staining showed a compact mass of epithelial cells in vehicle-treated mice, whereas RocA-treated tumors exhibited loose epithelial cell aggregates with a higher number of interspersed mesenchymal cells (Figure 6F, left). In addition, RocA treatment resulted in a 3.2-fold decrease of Ki-67-positive cells in tumor sections from RocA-treated mice compared with that in vehicle-treated mice (Figure 6F, middle and 6G, upper). Furthermore, we found a 4.1-fold decrease of cyclin D1-positive cells in tumor sections from RocA-treated mice relative to that in vehicle-treated mice (Figure 6F, right and 6G, lower). Therefore, RocA is a potent small molecule that suppresses the growth of AsPC-1 cell-derived tumors *in vivo*.

## Discussion

The RAS-RAF-ERK signaling pathway has been intensely researched because of its central role in cancer cell proliferation, survival, invasion, and metastasis [21,29,30]. However, the small G-protein RAS appears to be an intractable therapeutic target. Alternatively, downstream kinases in the pathway can be targeted, such as RAF and MEK. Although inhibitors of RAF and MEK have shown therapeutic value, tumor resistances counteract their effectiveness [31-33]. Therefore, targeting scaffold proteins such as PHB may be a valid downstream target of RAS.

Here, we represent a new strategy for combating oncogenic RAS-ERK signaling pathway by targeting the PHB-CRAF interaction in pancreatic ductal adenocarcinoma. Considering that PHB forms a signaling complex with CRAF to regulate RAF-MEK-ERK pathway, we demonstrated that PHB was highly expressed in human pancreatic cancer and depletion of PHB reduced in vitro invasion of RAS-driven cancer cells. In addition, we found that depletion of PHB suppressed ERK activity. Furthermore, ERK activity was blocked by RocA in RAS-driven cancer cells. RocA also suppressed the growth and invasion of these cells in vitro and inhibited the growth of tumor xenografts in SCID mice. Notably, no such effects were observed in normal epithelial cells, demonstrating the specificity of this response. To assess the consequences of long-term RocA treatment, we found that RocA extended the lifespan of these animals with a notable lack of toxicity compared with that of animals treated with the vehicle only.

Thus, RocA suppressed ERK activity and inhibited *in vitro* and *in vivo* growth and migration of cancer cells, which are dependent on the ERK pathway. These results indicated that the PHB scaffold function is essential in ERK pathway-driven pancreatic cancer cells and validated PHB as a therapeutic target. More importantly, RocA was relatively nontoxic in PHB-deficient cancer and normal cells, suggesting that the scaffold function of PHB in the ERK pathway is dispensable in these cells. These observations suggest that ERK-driven cancer cells are particularly sensitive to both the levels and fidelity of ERK signaling, and that PHB plays a key role in ensuring that signaling is maintained at optimal levels. This inference may be why these cells are sensitive to disruption between CRAF and PHB by RocA.

Although our work provides a strong case for targeting PHB by RocA, it remains to be determined whether this known RocA activity may contribute to the overall effect of RocA on survival of pancreatic tumor cells *in vivo* and *in vitro*.

RocA has been reported to inhibit translation initiation to block HSF1 activation by stimulating an interaction of RNA with eIF4A helicase [34]. However, the RAS-RAF-ERK pathway is a key pathway that regulates protein synthesis and tumor survival [35,36]. RocA does not directly disrupt the translational machinery, but it inhibits the ERK pathway to prevent eIF4E phosphorylation and subsequently suppress translation [37,38]. Therefore, the translation inhibition and the degree to which their roles overlap complement or antagonize each other in modulating the pathway remain elusive. Additionally, it is unclear if RocA will succumb to the same pitfalls as other RAF-targeting therapies. Clearly, unravelling the complexity of disrupting PHB function will be challenging. However, our study represents a compelling argument for future investigating PHB in oncogenic pathway as a drug target.

## Conclusions

In summary, targeting the PHB-CRAF interaction represents a potential new avenue for the treatment of pancreatic cancer. This new approach could be an important supplement therapy and may provide mechanistic insight into the molecular basis of RAS-RAF-ERK pathway in pancreatic cancer. Thus, RocA treatment as a new targeted therapy is a promising approach for improving the current therapeutic strategies and overcoming resistances of kinase inhibitors, and should be investigated in future preclinical and clinical studies.

## Methods

### Reagents

Antibodies against PHB, ERK1, CRAF, RAS, Ki-67, and cyclin-D1 were obtained from Abcam (Cambridge, UK). An anti-α-tubulin antibody was obtained from Santa Cruz Biotechnology (Santa Cruz, CA). EGF, an Epithelial Mesenchymal Transition Antibody Sampler Kit, and antibodies against phosphorylated forms of ERK1/2 and CRAF (Ser338) were purchased from Cell Signaling Technology (Danvers, MA). Cell culture reagents were purchased from GIBCO/Invitrogen (Carlsbad, CA). Specific siRNA against PHB and control siRNA were purchased from Qiagen (Valencia, CA). RocA (>98% pure) was procured from Enzo Life Sciences (Lörrach, Germany). All chemicals were purchased from Sigma Aldrich (St. Louis, MO) unless indicated otherwise.

### Cell lines, culture conditions, and clinical specimens

Pancreatic cancer cell lines AsPC-1, Capan-2, and Panc-1 were obtained from the American Type Culture Collection (Rockville, MD). The cells were cultured in RPMI 1640 medium supplemented with 10% fetal calf serum, penicillin (100 U/ml), and streptomycin (100 μg/ml) in a humidified incubator containing 5% CO_2_ at 37°C. The normal human breast epithelial cell line Hs 578Bst and normal human liver cell line L02 were purchased from Shanghai Cell Bank (Shanghai, China). These cells were cultured in Dulbecco's modified Eagle's medium supplemented with 10% fetal calf serum, penicillin (100 U/ml), and streptomycin (100 μg/ml) in a humidified incubator containing 5% CO_2_ at 37°C. AsPC-1 and Capan-2 cells were serum starved for 4–6 h before stimulation with EGF at a final concentration of 50 ng/ml for 15 min. Tissue samples were collected from patients during pancreatic resections for PDAC (n = 46). Normal pancreatic tissue samples were obtained through an organ donor procurement program when there was no suitable recipient for pancreatic transplantation (n = 11). Pancreatic tissues were immediately stored at –80°C or formalin-fixed and paraffin-embedded for histological analysis. The use of human tissue was approved by the local ethics committee (Tongji Medical College, China) and written informed consent was obtained from patients prior to surgery.

### RT-PCR and quantitative real-time PCR

At the indicated time points, total RNA was harvested from cells by treatment with TRIzol (Invitrogen) according to the manufacturer’s protocol. For RT-PCR analysis, total RNA (1 μg) was used as a template for cDNA synthesis with a reverse transcription kit (Fermentas). Equal amounts of cDNA were used in PCR analyses. The following primers were used in this study. PHB: forward, 5′-CTGCCTTATATAATGTGGATGCTG-3′ and reverse, 5′-GCTCTCTCTGGGTGATTAGTTCTC-3′; β-actin: forward, 5′-AGTGTGACGTCGACATCCGC-3′ and reverse, 5′-GACTCGTCGTACTCCTGCTT-3′. For quantitative real-time PCR analysis, the relative amount of PHB mRNA was determined using a Quantitect™ SYBR® Green RT-PCR Kit (Qiagen) following the manufacturer’s instructions. The expression level of PHB mRNA was normalized against the internal standard, GAPDH. The following primers were used in the analyses. PHB: forward, 5′-CTTTGACTGCCGTTCTCGAC-3′ and reverse, 5′-TGGGTGGATTAGTTCTCCAGC-3′; GAPDH: forward, 5′-GGTATCGTGGAAGGACTCATGACGA-3′ and reverse, 5′-ATGCCAGTGAGCTTCCCGTTCAG-3′.

### Immunoprecipitation and immunoblot analysis

Cells were washed twice with ice-cold PBS and then lysed with ice-cold lysis buffer. Lysates were kept on ice for 30 min and then centrifuged at 17,000 *g* for 15 min at 4°C. Equal amounts of proteins were used for immunoprecipitation of PHB by overnight incubation (at 4°C with gentle rocking) with specific antibodies and then protein G-agarose. The agarose beads were washed five times with washing buffer, resuspended in 2× Laemmli buffer, and then boiled for 5 min. For western blotting analysis, equal amounts of proteins were separated by SDS-polyacrylamide gel electrophoresis and then transferred onto polyvinylidene difluoride membranes (Millipore). The membranes were blocked with 5% bovine serum albumin (BSA) in Tris-buffered saline-Tween 20 for 2 h and then incubated with primary antibodies at 4°C overnight. Immunoreactive proteins were detected with horseradish peroxidase-conjugated secondary antibodies.

### Confocal microscopy

Cells were fixed with 4% formaldehyde in PBS for 10 min, washed, and then permeabilized with 0.5% Triton X-100 for 15 min. The fixed cells were incubated with 1% BSA in PBS for 60 min and then overnight with gentle rocking at 4°C with antibodies against PHB and p-ERK1/2. The cells were washed five times with 1% BSA and then incubated for 50 min with Alexa Fluor 647-labeled rabbit anti-mouse IgG (H + L) (Abcam) to detect PHB and Alexa Fluor 488-labeled goat anti-rabbit IgG (H + L) (Abcam) to detect p-ERK1/2. Nuclei were counterstained with DAPI. After washing the cells with PBS and mounting with SlowFade Antifade Kit (Life Technologies), confocal images were obtained with an FV-1000 confocal laser scanning microscope (Olympus).

### PHB knockdown

Cells were transfected with nonsense siRNA or siRNA targeting PHB (5′-CAGAAA UCACUGUGAAAUUTT-3′) using HiPerFect transfection reagent (Qiagen) according to the manufacturer’s protocol. Cells were collected at the indicated time points after transfection for various assays.

### CCK-8 assay

Cells were seeded in 96-well plates at 5 × 10^3^ cells per well and allowed to adhere for 24 h at 37°C. The cells were then treated with RocA (100 nM) or DMSO for 16 h. Following the treatments, a CCK-8 solution (10 μl; Dojindo, Kumamoto, Japan) was added to each well. After incubation at 37°C for another 2 h, viable cells were detected by measuring the absorbance at 570 nm using an EL×800 Absorbance Microplate Reader (Bio-Tek, Seattle, WA, USA). Cell viability was expressed as the percentage absorbance of cells treated with RocA compared with that of DMSO-treated cells.

### Transwell migration assay

Cell migration was analyzed using a modified two-chamber transwell system (BD) following the manufacturer’s instructions. Cells were detached by trypsin-EDTA, washed once with serum-free medium, and then resuspended in serum-free medium. Then, 0.5 ml of either complete culture medium or serum-free medium containing 50 ng/ml EGF was added to each lower chamber. Cells (1 × 10^5^) were added to each transwell insert and allowed to migrate for 12 h a 37°C. The cells on the upper surface of the transwells were removed using cotton swabs. Migrated cells attached on the undersurface were fixed with 4% paraformaldehyde for 10 min and then stained with a crystal violet solution (0.5% in water) for 10 min. Cells were counted under a microscope at 200× magnification.

### Subcutaneous and orthotopic xenografts in SCID mice

SCID mice were purchased from HFK Bioscience Ltd (Beijing, China). Animal experiments were performed in accordance with relevant institutional and national regulations, and research protocols were approved by the relevant authorities. AsPC-1 cells (3 × 10^6^) suspended in a 100 μl mixture of equal volumes of medium and matrigel were implanted subcutaneously into the right flank of 6-week-old female SCID mice. When the tumors had reached a volume of about 50-70 mm^3^, the mice were then randomly divided into two groups. The treatment group received an intraperitoneal injection of RocA (5.0 mg/kg in 80 μl olive oil, n = 5), whereas the vehicle control group received olive oil alone (n = 6). These treatments were carried out once daily for 48 days. Tumor volumes and the body weight of animals were measured twice a week. Tumor volumes (mm^3^) were calculated with the following formula: V = LS^2^/2 (where L is the longest diameter and S is the shortest diameter). At the end of experiment, the mice were sacrificed and the tumors were harvested, fixed in formalin, and embedded in paraffin for tissue sectioning and immunohistochemistry. For orthotopic metastasis assays, AsPC-1 cells (3 × 10^6^) were orthotopically injected into the pancreas of mice (n = 6) as described previously [39]. At 1 week post-implantation, RocA (5.0 mg/kg in 80 μl olive oil, n = 3) or the vehicle (1% DMSO in olive oil, n = 3) was administrated via intraperitoneal injection daily for 3 weeks. Then, these mice were sacrificed to evaluate metastasis to the organs such as the liver and lung. The metastatic nodules in the right lung and liver were quantified under a dissecting microscope. Another ten mice were subjected to the same treatment. The survival time of these mice in each group (RocA-treated group, n = 5; Vehicle control group, n = 5) was monitored.

### Immunohistochemistry

Immunohistochemical analysis was performed as described previously [40] with antibodies against PHB, Ki-67, and cyclin-D1.

### Statistics

Data are representative of at least three independent experiments or multiple independent mice as indicated. Statistical analyses were performed by Student’s *t-*tests and analysis of variance followed by post-hoc comparisons. Kaplan-Meier survival data were reanalyzed using the log-rank test.

## Competing interests

The authors declare that they have no competing interests.

## Authors’ contributions

ZL and YH carried out most of the experiments; MA participated in the experiments and performed the statistical analysis; ZSC and FH participated in the design of the study and helped draft the manuscript. All authors read and approved the final manuscript.

## Supplementary Material

Additional file 1: Figure S1 Cell viability and migration of AsPC-1 and Capan-2 cells. **Figure S2.** Expression of PHB mRNA and protein in AsPC-1 and Capan-2 cells. **Figure S3.** Expression of PHB in human normal pancreas and PDAC. **Figure S4.** Expression of PHB mRNA and protein in siCon- and siPHB-treated pancreatic cancer cells. **Figure S5.** Effect of RocA on the proliferation of Capan-2 cells. **Figure S6.** Effect of RocA on the survival rate of AsPC-1 cells implanted in the pancreas of the mice. Click here for file
