# Supplementary material for: Targeting the prohibitin scaffold-CRAF kinase interaction in RAS-ERK-driven pancreatic ductal adenocarcinoma
Source: Mol Cancer. 2014 Feb 25;13:38. doi: 10.1186/1476-4598-13-38 (PMC3938031; doi:10.1186/1476-4598-13-38)

***Supplemental Data***

**Fig. S1 Cell viability and migration of AsPC-1 and Capan-2 cells**

**A.** Viabilities of AsPC-1 and Capan-2 cells were determined by CCK-8 assays. The plot shows the quantification of cell viability relative to AsPC-1 cell viability that was set to 100%. Values are the means ± SD of triplicate samples, *P < 0.01. **B**. Transwell migration assays of AsPC-1 and Capan-2 cells. The plot shows the quantification of cells that passed through the filters relative to AsPC-1 cell migration that was set to 100%. Values are the means ± SD of triplicate samples, *P < 0.01. Scale bars, 25 μm.


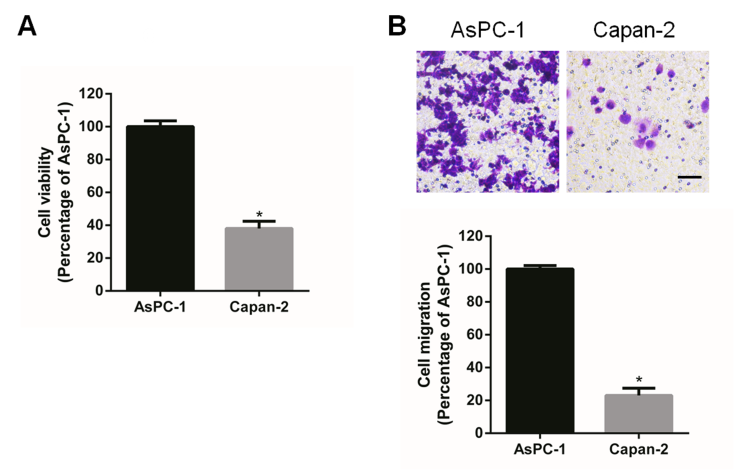


**Fig. S2 Expression of PHB mRNA and protein in AsPC-1 and Capan-2 cells**

**A**. Quantification of PHB mRNA in AsPC-1 and Capan-2 cells by RT-PCR. Values are the means ± SD of triplicate samples, *P < 0.01. **B**. Quantification of PHB protein in AsPC-1 and Capan-2 cells by immunoblot analysis. Values are the means ± SD of triplicate samples, *P < 0.01.


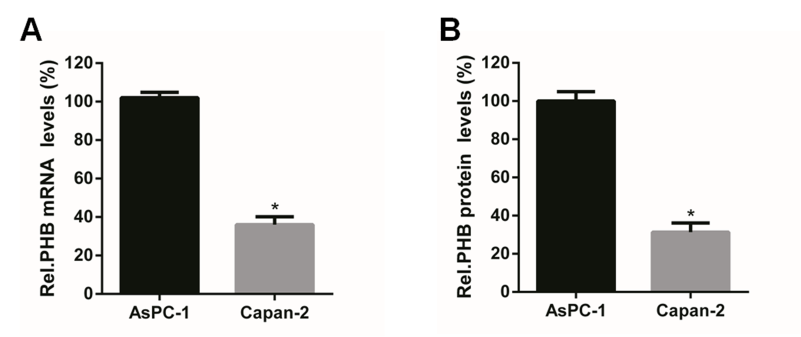


**Fig. S3 Expression of PHB in human normal pancreas and PDAC**

Quantification of PHB expression in normal pancreas and PDAC tissue. Tumors were blindly scored based on the strength of PHB staining (n=11 normal tissue samples and n=46 PDAC tissue samples.

PHB Stain

weak moderate strong

Normal 7 (63.6%) 4(36.4%) 0 (0%)

PDAC 5(10.9%) 14 (30.4%) 27 (58.7%)

**Fig. S4 Expression of PHB mRNA and protein in siCon- and siPHB-treated pancreatic cancer cells**

AsPC-1 and Panc-1 cells were transfected with PHB-specific (siPHB) or control (siCon) siRNAs for 48 h. Total mRNA was then collected and analyzed by quantitative real-time PCR. Quantification of PHB mRNA levels in PHB-specific (siPHB)- or control siRNA (siCon)-treated AsPC-1 (**A**) and Panc-1 (**B**) cells. Values are the mean ± SD of triplicate samples, *P < 0.01.


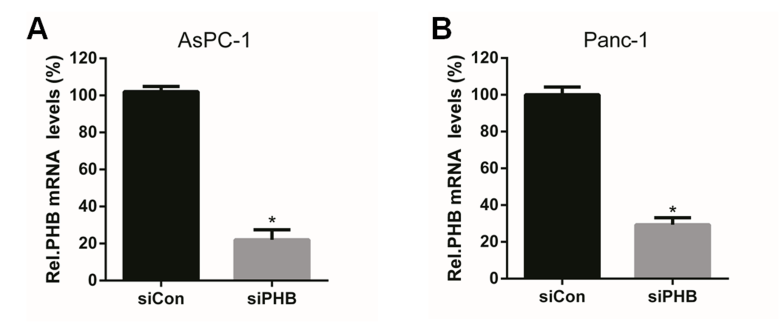


**Fig. S5 Effect of RocA on the proliferation of Capan-2 cells**

Capan-2 cells were treated with RocA (100 nM) or DMSO for 16 h and then cell viability was determined by CCK-8 assays. Values are the means ± SD of triplicate samples.


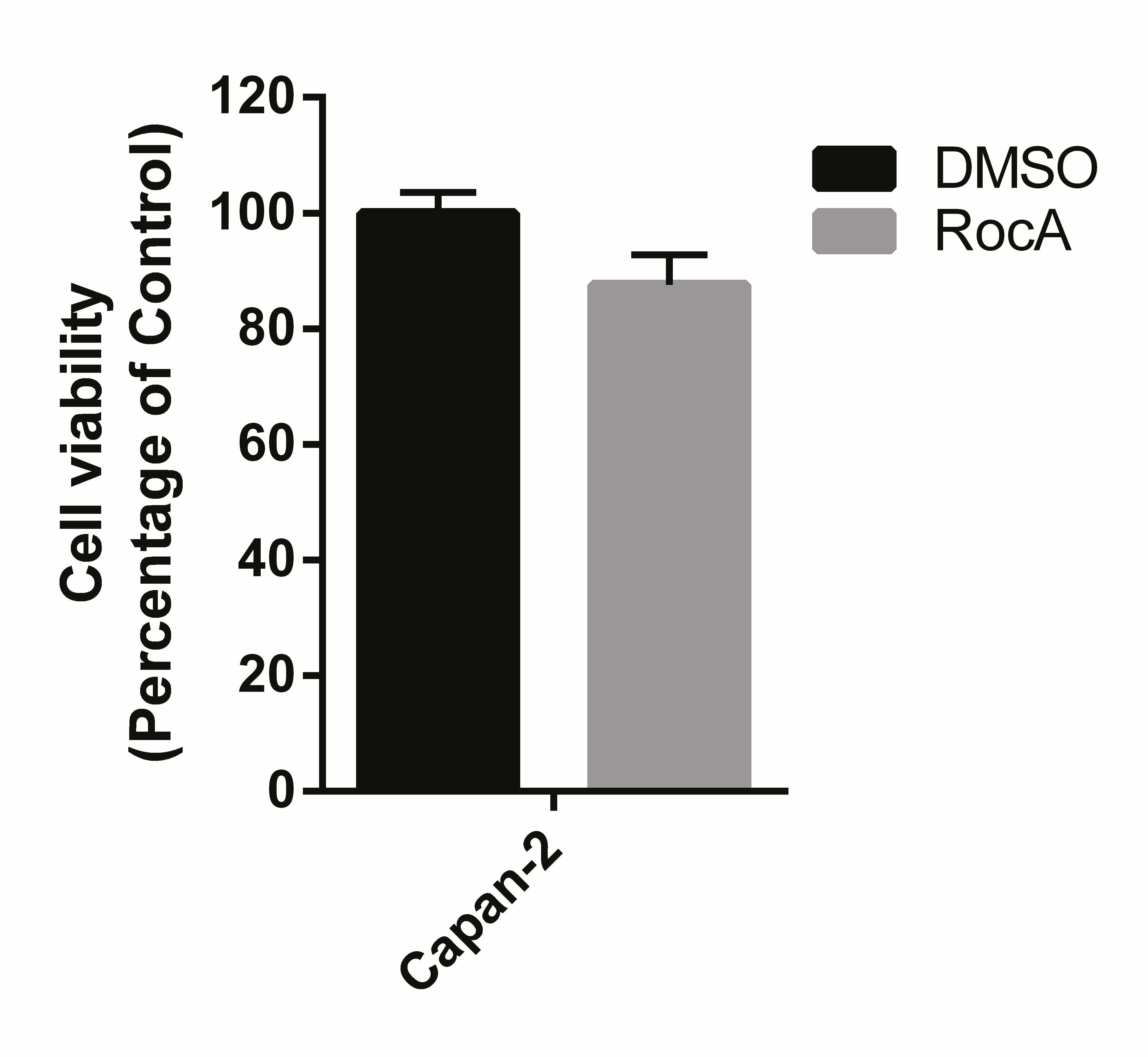


**Fig. S6 Effect of RocA on the survival rate of AsPC-1 cells implanted in the pancreas of the mice**

AsPC-1 cells (3×10^6^) were orthotopically injected into the pancreas of mice (n=10) as described in method. At 1 week post-implantation, RocA (5.0 mg/kg, n=5) or the vehicle (1% DMSO in olive oil, n=5) was administrated via intraperitoneal injection daily. The survival time of these mice in each group was monitored. **A**. Survival of mice with established tumor burden randomized to receive RocA or vehicle by intraperitoneal injection. **B**. Median survival of the mice in (**A**). Statistical significance was calculated by the log-rank test. Data are shown as the means ± SD.


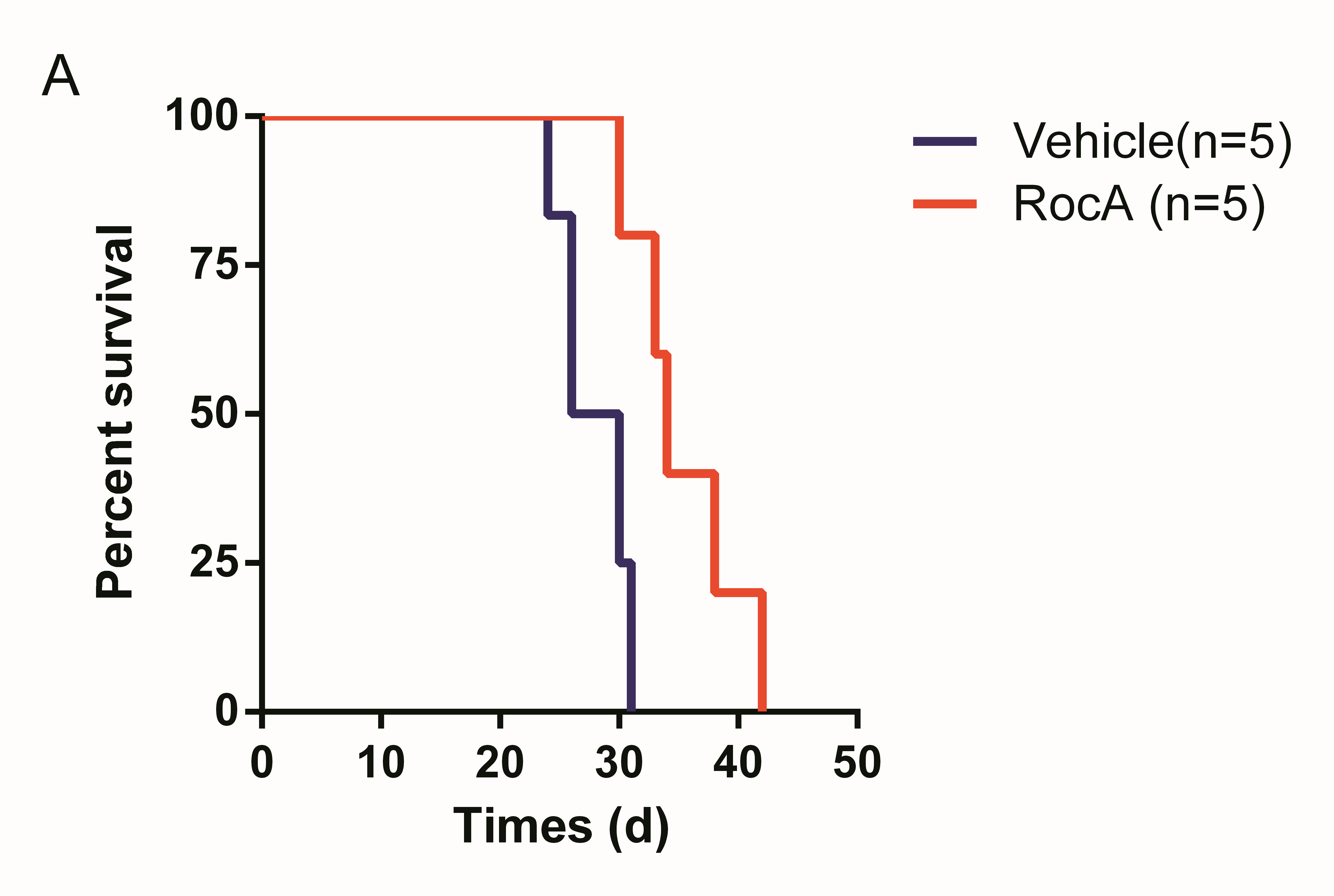

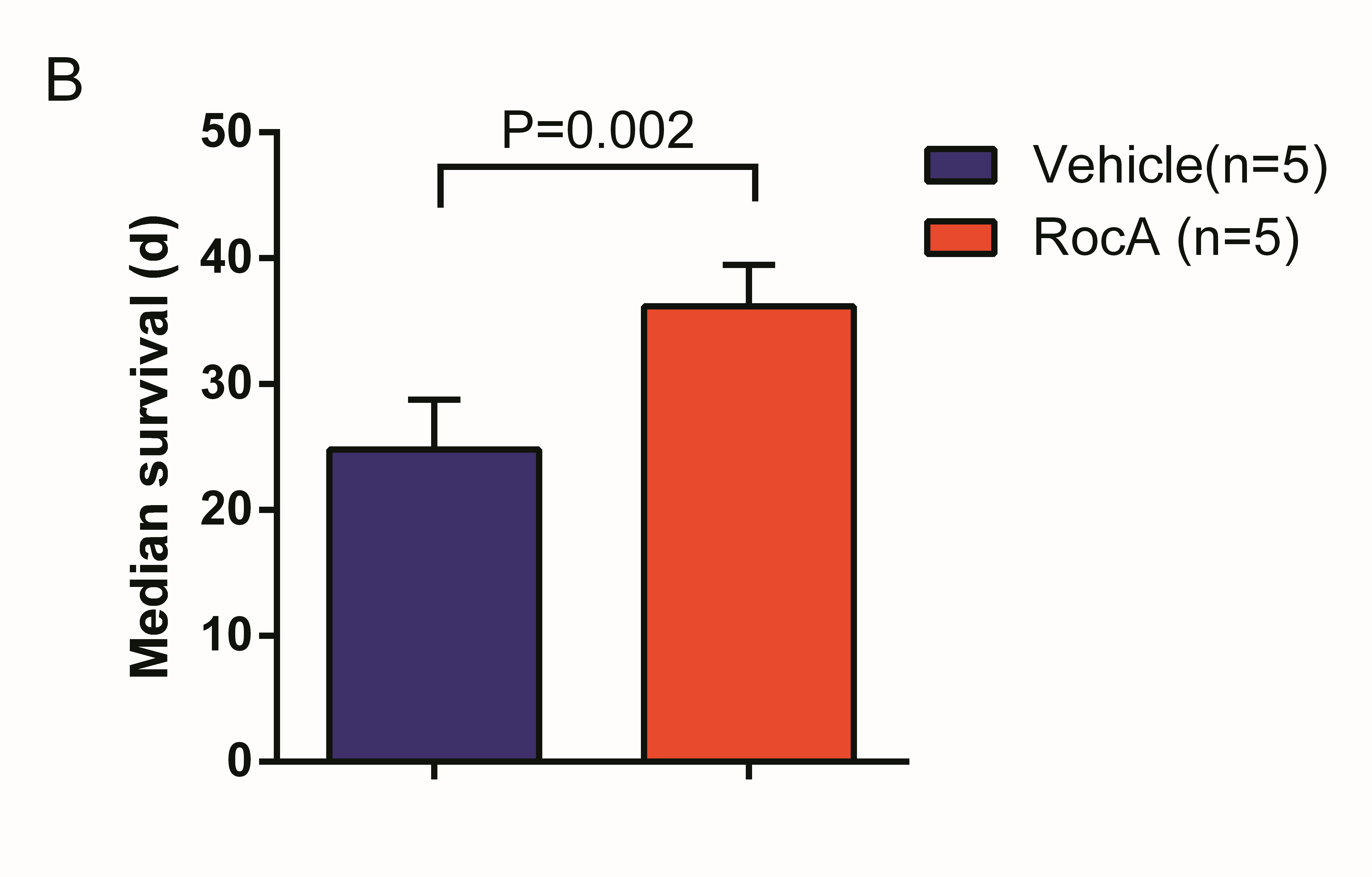

Supplement: Additional file 1: Figure S1 — Cell viability and migration of AsPC-1 and Capan-2 cells. Figure S2. Expression of PHB mRNA and protein in AsPC-1 and Capan-2 cells. Figure S3. Expression of PHB in human normal pancreas and PDAC. Figure S4. Expression of PHB mRNA and protein in siCon- and siPHB-treated pancreatic cancer cells. Figure S5. Effect of RocA on the proliferation of Capan-2 cells. Figure S6. Effect of RocA on the survival rate of AsPC-1 cells implanted in the pancreas of the mice. [file 1476-4598-13-38-S1.doc]
